# "Because Even the Person Living With HIV/AIDS Might Need to Make Babies" – Perspectives on the Drivers of Feasibility and Acceptability of an Integrated Community Health Worker Model in Iringa, Tanzania

**DOI:** 10.15171/ijhpm.2019.38

**Published:** 2019-06-11

**Authors:** Katharine D. Shelley, Gasto Frumence, Rose Mpembeni, George Mwinnyaa, Juliana Joachim, Hawa Kadria Kisusi, Japhet Killewo, Abdullah H. Baqui, David H. Peters, Asha S. George

**Affiliations:** ^1^Department of International Health, Johns Hopkins Bloomberg School of Public Health, Baltimore, MD, USA.; ^2^Department of Development Studies, School of Public Health and Social Sciences, Muhimbili University of Health and Allied Sciences, Dar es Salaam, Tanzania.; ^3^Department of Epidemiology and Biostatistics, School of Public Health and Social Sciences, Muhimbili University of Health and Allied Sciences, Dar es Salaam, Tanzania.; ^4^Christian Social Services Commission, Dar es Salaam, Tanzania.; ^5^School of Public Health, University of the Western Cape, Cape Town, South Africa.

**Keywords:** Community Health Workers, Implementation Research, Integration, Tanzania, Workload

## Abstract

**Background:** Countries with health workforce shortages are increasingly turning to multipurpose community health workers (CHWs) to extend integrated services to the community-level. However, there may be tradeoffs with the number of tasks a CHW can effectively perform before quality and/or productivity decline. This qualitative study was conducted within an existing program in Iringa, Tanzania where HIV-focused CHWs working as volunteers received additional training on maternal, newborn, and child health (MNCH) promotion, thereby establishing a dual role CHW model.

**Methods:** To evaluate the feasibility and acceptability of the combined HIV/MNCH CHW model, qualitative in-depth interviews (IDIs) with 36 CHWs, 21 supervisors, and 10 program managers were conducted following integration of HIV and MNCH responsibilities (n=67). Thematic analysis explored perspectives on task planning, prioritization and integration, workload, and the feasibility and acceptability of the dual role model. Interview data and field observations were also used to describe implementation differences between HIV and MNCH roles as a basis for further contextualizing the qualitative findings.

**Results:** Perspectives from a diverse set of stakeholders suggested provision of both HIV and MNCH health promotion by CHWs was feasible. Most CHWs attempted to balance HIV/MNCH responsibilities, although some prioritized MNCH tasks. An increased workload from MNCH did not appear to interfere with HIV responsibilities but drew time away from other income-generating activities on which volunteer CHWs rely. Satisfaction with the dual role model hinged on increased community respect, gaining new knowledge/skills, and improving community health, while the remuneration-level caused dissatisfaction, a complaint that could challenge sustainability.

**Conclusions:** Despite extensive literature on integration, little research at the community level exists. This study demonstrated CHWs can feasibly balance HIV and MNCH roles, but not without some challenges related to the heavier workload. Further research is necessary to determine the quality of health promotion in both HIV and MNCH domains, and whether the dual role model can be maintained over time among these volunteers.

## Background

HIV programs are often organized and delivered as stand-alone services (“vertical”) in sub-Saharan Africa.^1,2^ However, integration of maternal, newborn, and child health (MNCH), family planning, and HIV services is recognized as an important strategy for reducing maternal and child mortality, and the Global Plan for Elimination of Mother-to-Child-Transmission of HIV calls for “leveraging synergies, linkages, and integration for improved sustainability.”^3^ Terms are used interchangeably with integration (*coordination*, *linkage*, *collaboration*, *alignment*, *networks)*, along with a variety of concepts (*integrated care*, *integrated health services*, *coordinated care, continuum of care,* and *integrated delivery networks)*,^4^ underscoring the numerous definitions and interpretations of what constitutes “integration.”^5^ Integration is intended to produce efficiency gains, reduce fragmentation of services, increase access to health services, increase patient satisfaction, and improve health outcomes.^4,6^ While there is some evidence from low- and middle-income country contexts indicating increased service utilization following the addition of services, improvements in health outcomes have generally been limited.^6^ Findings from a recent systematic review suggest sufficient evidence that HIV integration with MNCH, family planning, and nutrition was feasible to implement, with some studies documenting improvements in health coverage and outcomes; however, evidence for HIV-MNCH integration at the community-level was largely absent as integration research has tended to focus on facility-based contexts.^3^

A multipurpose, paid community health worker (CHW) model has gained policy momentum in many Sub-Saharan African countries for reasons of cost, efficiency, access, and benefits of integration. However, there may be tradeoffs with the number of tasks a CHW can effectively perform before quality and productivity begin to decline from work overload. In Rwanda, adding family planning provision to the existing responsibilities of a nationally-supported volunteer CHW program did not significantly change time spent on service provision or travel, and nearly all CHWs reported workload manageability and high job satisfaction.^7^ In Bangladesh, adding treatment for severe acute malnutrition to the workload of CHWs focused on case management of acute respiratory infection and diarrhea led CHWs to work significantly more hours per week, while maintaining quality of care for both preventive and curative tasks.^8^ However, in Malawi, a qualitative study identified several challenges to integrated responsibilities for CHWs: task overloading (CHWs unable to fulfill multiple roles); task specialization (over-emphasis on newly learned skills); and difficulty managing competing disease priorities (multiple programs and stakeholders).^9^

In Tanzania, the health system is supported by an extensive network of about 41 000 CHWs, mainly volunteers in vertically-oriented programs, with nearly half centered on either HIV or MNCH services.^10^ However, the lack of coordination and harmonization across these numerous CHW cadres, supported by implementing partners and non-governmental organizations, has prompted Tanzania to begin establishing a multipurpose, national cadre of paid CHWs.^12^ To support Tanzania’s development and rollout of an integrated CHW cadre, this study sought to describe a program innovation to combine HIV and MNCH health promotion at the community-level and qualitatively explore *how* CHWs manage their dual role responsibilities and *why* it worked (or not).

## Methods

### Program Context

TUNAJALI (Swahili for “We Care”) was a large-scale initiative funded over two 5-year cycles (2006-2011; 2012-2017) by the United States Agency for International Development (USAID) to prevent HIV/AIDS and increase access to HIV care, treatment and support services in Tanzania, focusing on both community and facility-based services.^13^ TUNAJALI II was implemented by Deloitte Consulting Limited (prime partner) and Christian Social Services Commission (technical partner) across 5 regions of Tanzania, namely Dodoma, Iringa, Njombe, Morogoro, and Singida. This study focuses on TUNAJALI II’s community-based program activities in Iringa Region (2012 population: 941 238), located in the Southern Highlands zone of mainland Tanzania, in the districts of Kilolo (2012 population: 218 130) and Iringa Rural (2012 population: 254 032).^14^ The prevalence of HIV in Iringa Region (9.1%) ranks second highest nationally and is nearly double the national average (5.1%).^15^ However, coverage estimates for MNCH indicators were higher in Iringa Region than nationally in the 2004-2005, 2010, and 2015 Demographic and Health Surveys – for example, the proportion of live births delivered at health facilities has improved over time both in Iringa (71.8%, 80.4%, to 92.9%) as well as nationally across Tanzania (47.1%, 50.1%, to 62.6%) although remains comparatively lower.^16-18^ Through TUNAJALI II, local civil society organizations (CSOs) managed over 400 volunteer CHWs in 4 Iringa districts. These village-based CHWs received 12 days of training and are tasked with providing home-based HIV services, following 15 to 100 households each, with a major focus on treatment adherence, retention, and palliative support (Box 1).^19^ In recent years, the HIV workload of CHWs has decreased as antiretroviral drugs became more available and the number of extremely sick HIV/AIDS patients declined. This led program administrators to suggest that HIV-focused CHWs could absorb additional duties involving MNCH to increase antenatal care (ANC) utilization and health facility deliveries. Therefore, during 2015 approximately half the HIV CHWs working with this program in Iringa Region were trained for 3 weeks using the MNCH curriculum approved by Tanzania’s Ministry of Health, with health promotion topics organized around the recommended timing and frequency of home visits (Box 1).^20^ These “dual role” CHWs were tasked with both HIV and MNCH services (compared to “single role” CHWs focused on HIV services only).

**Box 1. CHW Health Promotion Counseling Topics and Tasks**

* HIV/AIDS*

- Tracking HIV clients who miss appointments
- Treatment adherence
- Psychosocial support
- Referral to facility for opportunistic infections
- Referral for social and economic services
- Sensitization on voluntary HIV counseling and testing
- Condom distribution
- Stigma reduction advocacy
- Palliative care for pain control and comfort
- Good hygiene and nutrition

* 
Maternal, Newborn and Child Health*

- ANC booking, pregnancy danger signs, facility delivery, birth preparedness, breastfeeding
- HIV prevention, including prevention of mother-to-child transmission of HIV
- Malaria prevention
- Newborn care, danger signs, infection prevention
- Family planning and postpartum care

Abbreviations: CHW, community health worker; ANC, antenatal care.

A facility-based HIV healthcare worker provided HIV-focused supervision to both single and dual role CHWs. However, dual role CHWs also reported to a second facility-based healthcare worker for MNCH-related supervision. “Home based care (HBC) focal persons” from the CSO also provided monthly HIV-focused supervision to CHWs. Modest monthly stipends were provided through TUNAJALI II as remuneration to CHWs, which increased from the initial US$17 to US$20 for all CHWs following MNCH training, regardless of whether they were assigned to single or dual responsibilities.

This study is part of a larger mixed methods implementation research evaluation of the dual role CHW model. Qualitative and quantitative data were collected from February 2016 to January 2017, with concurrent analysis and triangulation of mixed methods data from June 2016 to March 2017 (Supplementary file 1). Here we present qualitative findings on feasibility and acceptability, with reference to quantitative data in associated publications.

### Sampling

**Figure 1:**
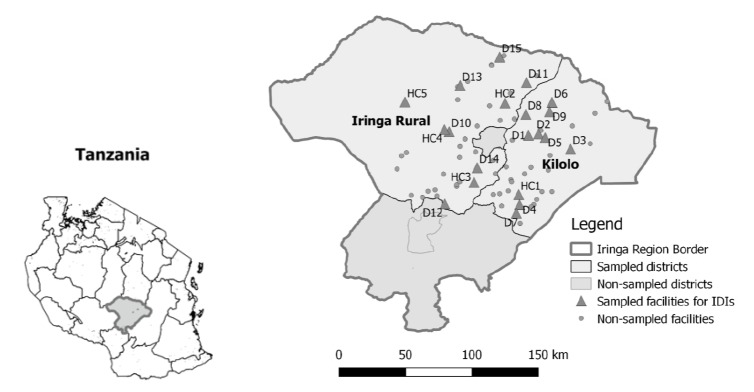
Map of Iringa Region in the Southern Highlands of Tanzania and Location of the 20 Sampled Facilities in Iringa Rural and Kilolo Districts.
Notes: Dispensaries: D1 to D15; Health Centers: HC1 to HC5. Maps were generated using QGIS Version 2.18.^21^ Country, regional and district boundaries were sourced from Tanzania’s National Bureau of Statistics.^22^ Coordinates for health facilities were sourced from online health facility registry maintained by Tanzania’s MoHCDGEC.^23^

In Iringa Rural and Kilolo, 6 types of respondents were selected for interviews using several sampling approaches (Table 1). At the prime partner’s regional office, the director, HIV technical officer, and reproductive and child health technical officer were interviewed. At the CSOs, all staff members with program responsibilities were interviewed, including the director, project coordinators, and HBC focal persons. CHWs and their supervisors were interviewed from 20 facilities (10 per district) among the 71 facilities involved across the two districts. Purposive sampling methods were used to achieve maximum variation in facility and CHW characteristics, including: facility type (health center and dispensary) and ownership (public, private faith-based); location; availability of HIV care and treatment services; monthly service utilization for ANC and deliveries; number of CHWs per facility; type of CHWs (single vs. dual role); and Gender of CHWs. The locations of sampled dispensaries and health centers are presented in Figure 1 (notably clustered toward the center owing to the region’s geography with Ruaha National Park to the West and Udzungwa Mountains National Park to the East).

**Table 1:** Summary of Total IDIs Conducted and Sampling Approach for Each Respondent Group

**Group**	**Respondent Type**	**IDIs**	**Sampling Approach**
CHWs	Single role CHWs	15	CHWs linked to purposively sampled facilities for maximum variation
	Dual role CHWs	21	
Supervisors	HIV supervisors at facility	10	Supervisors from purposively sampled facilities for maximum variation
	MNCH supervisors at facility	11	
Managers	CSO staff	7	“Focal persons,” program managers, and/or technical staff
	Prime partner staff	3	
	**Total Interviews**	**67**	

Abbreviations: IDI, in-depth interview; CHWs, community health workers; CSO, civil society organization; MNCH, maternal, newborn, and child health.

### Data Collection

Semi-structured in-depth interviews (IDIs), 30-60 minutes long, were conducted in Swahili by 6 trained Tanzanian research assistants. IDI topic guides were organized by several *a priori* themes: Training and guidance for integration of HIV-MNCH tasks; CHW role expansion from HIV to MNCH; CHW performance; workload balance; feasibility and acceptability; and recommendations for improvement. Interviews were conducted over 3 weeks during February 2016, early in the implementation phase: 3 months after MNCH training in Iringa Rural and 8 months after MNCH training in Kilolo. The CSOs convened regularly scheduled monthly meetings with CHWs at central locations to share information, discuss challenges, collect monthly HIV summary reports, and assess data quality. Where possible, the research team scheduled CHW interviews to coincide with these monthly meetings. Visits to selected facilities were scheduled to interview supervisors, as well as CHWs not reached during the monthly meetings. Following each data collection event, the research team held debriefing sessions with the team leaders on main points given by respondents and emergent ideas and themes. Research assistants also submitted a form with summary notes on each IDI, including interview setting and quality, key summary points, and any new information. In addition, information from program documentation (eg, quarterly reports) and field observations (eg, monthly CSO meetings and visits to facilities, district health offices, and CSO officers) was compiled to further inform understanding of HIV and MNCH roles and program supervision and oversight.

### Data Management and Analysis

Audio-recorded interviews were transcribed by 6 research assistants, 3 of whom had conducted the IDIs. Personal identifiers were removed from transcripts to ensure confidentiality. To assess transcription quality, a Tanzanian research scientist listened to audio-recorded interviews while reading along with the transcript for 2 files per transcriptionist. Interviews were translated from Swahili into English. Translation quality was assessed using the first and seventh documents submitted by each translator, reading each Swahili paragraph followed by the English translation for the full document.

Qualitative data management, coding, and thematic analysis were performed using the web-based software, Dedoose.^24^ Familiarization with field notes and interview transcripts supported the initial preliminary coding structure, based on presumptive topics in the interview guides and emerging themes identified during the familiarization phase. The structure of the preliminary codebook was independently tested by two primary coders experienced with CHW programs. Each coder deductively applied codes from the preliminary codebook to two selected transcripts. They then met to compare code agreement, examining line-by-line for discrepancies. Through this process, the coders reached consensus by discussing modifications to code definitions and agreeing on the definition of newly emergent codes. Four additional transcripts were independently coded and compared to assess agreement. Codes were further refined and added or deleted as new themes became apparent.^25^ The final codebook contained 68 codes within 6 thematic areas. Remaining transcripts were divided between the two primary coders.

Using a thematic analysis approach, code report excerpts were organized in data display matrices to chart key findings and illustrative quotes by IDI respondent type, including descriptive, text-based summaries for each key finding. Data matrices helped identify recurrent patterns and themes and facilitated comparison of diverse perspectives by respondent type. Key findings are presented in the context of understanding program feasibility and acceptability,^26^ using a subset of codes (Table 2). Interview data, field observations, and program documents were used to triangulate and compare implementation features of the HIV and MNCH roles.

**Table 2:** Codes Used in Analysis of Feasibility and Acceptability of the Integrated HIV-MNCH Model

**Themes**	**Sub-Themes**	**Codes**
Feasibility	Role expansion: HIV to MNCH	Task planning
		Task balance vs. prioritization
		Task integration
	Workload	Catchment change
		Workload time
		HIV role maintenance
		Integration challenges
Acceptability	Acceptability integrated model	CHW satisfaction with expanded role
		One Role vs. Two Roles for CHWs
		Advantages/disadvantages for CHWs
		Acceptability (by supervisors, CSO staff, and prime partner staff)

Abbreviations: MNCH, maternal, newborn, and child health; CHWs, community health workers; CSO, civil society organization.

## Results

### Sample Characteristics

A total of 67 IDIs were conducted with 6 respondent types: 21 dual role CHWs, 15 single role CHWs, 10 HIV supervisors, 11 MNCH supervisors, 7 program management staff from CSOs, and 3 technical staff from the prime partner (Table 1). CHWs and facility-based supervisors were drawn from 20 facilities (10 per district), including 15 dispensaries and 5 health centers (Figure 1). Most facilities were government-owned and roughly half provided HIV care and treatment services. Seven facilities were supported by single role CHWs, 12 facilities by dual role CHWs, and 1 facility was a “combination” site to which one single and one dual role CHW both reported. Service volume in almost all sampled facilities averaged less than 10 first ANC visits and less than 10 facility deliveries per month, per 2015 routine health information system data (see Supplementary file 2 for facility, CHW, and supervisor characteristics among interview respondents from each sampled facility).

On average, CHWs were 42.9 (±6.6) years old with 5.6 (±1.9) dependents and reported 8.7 (±2.8) years of community health experience. Half of CHWs were female (53%), majority were married (78%), and nearly all primary school (Standard 7) educated (97%). About 20% of CHWs reported a monthly income of less than US$25, meaning their income was mostly supported by the volunteer stipend – for context, Tanzania’s per capita gross national income in 2015 ($920) equates to a monthly income of $77.^27^ All CHWs (100%) reported agricultural farming and most (86%) also reported livestock keeping as a source of additional income. The median distance between a CHW’s home and supervisory health facility was 2 KM (IQR: 1–5). Most CHWs (72%) reported walking as their primary mode of travel to household visits and 33% reported travel time to the supervisory facility of more than one hour. CHWs were primarily linked to dispensaries (81%) for supervision and data reporting.

### HIV and MNCH Implementation: Key Differences

Some aspects of program implementation varied with HIV and MNCH tasks (Table 3). Interview respondents commonly referred to HIV-focused CHWs as “HBCs providers,” a volunteer cadre officially recognized by the Government of Tanzania to provide HIV support services. HBCs who received additional MNCH training were commonly called “*Wawezeshaji wa Afya ya Jamii* (WAJAs)” in Swahili, which translates to “Community Health Agents,” implying MNCH-focused tasks were synonymous with “community health” tasks.

**Table 3:** Comparison of Implementation Features for HIV and MNCH Tasks by CHWs

**Feature**	**HIV-Focused Role**	**MNCH-Focused Role**
Name	HBCs providers	WAJAs or “community health agents”
Novelty	Longstanding activity in the community	New health education/promotion activity in the community
Target	HIV-positive clients	Pregnant and postpartum women, newborns, and children under-5 years old
Content	Depending on individual client needs or group needs	Depending on visit timing (pregnancy vs. postpartum, child’s age)
Duration	Shorter visits for routine clients, longer visit for newly identified HIV-positive	Longer visits (up to 1 hour) for mothers and children
Setting	Individual homes or group settings in community	Individual homes
Stigma	Potential stigma with single role CHWs	Reduced stigma with expanded MNCH role (neighbors cannot distinguish visit purpose)
Urgency	Stable/chronic, less urgency	Potential for acute high-risk situations (obstetric or child illness), more urgency
Supervision	Facility-based HIV supervisor	Facility-based MNCH supervisor
CSO	“Focal person” from CSO provides HIV supervision	No MNCH supervision from CSO
Reporting^a^	HIV monthly report submitted to CSO and facility-based HIV supervisor	MNCH monthly report submitted to facility-based MNCH supervisor *only*

Abbreviations: HBC, home-based care; MNCH, maternal, newborn, and child health; CHWs, community health workers; CSO, civil society organization; WAJAs, *Wawezeshaji wa Afya ya Jamii*.

The longstanding HBC HIV program dates to 2006 in Iringa, whereas the MNCH role was introduced in mid-2015, bringing new health education topics to the community. The catchment area for dual role CHWs remained the same, but specific target populations differed. HIV tasks were designed to focus on HIV-positive individuals linked to CHWs for ongoing village/community-based support, whereas MNCH tasks were meant for pregnant and postpartum women and children under-5 years old identified through a village census. Target populations overlap somewhat: pregnant and postpartum women should receive education on both HIV and MNCH topics, in addition to HIV-positive clients (women, partners, and children) needing MNCH care.

Both HIV and MNCH roles emphasize prevention, with client interaction centering on health education and promotion, and CHWs helping to mobilize clients to health facility visits. HIV lessons cover general HIV prevention education messaging or individual client needs, often dependent on health status, duration of the disease, and past care. Sometimes, CHWs conduct group sessions for HIV clients, in lieu of individual household visits. In contrast, MNCH lessons vary with the timing of household visits and tend to require more time to cover topics involving pregnancy, newborns, and other children in the household.

**Figure 2:**
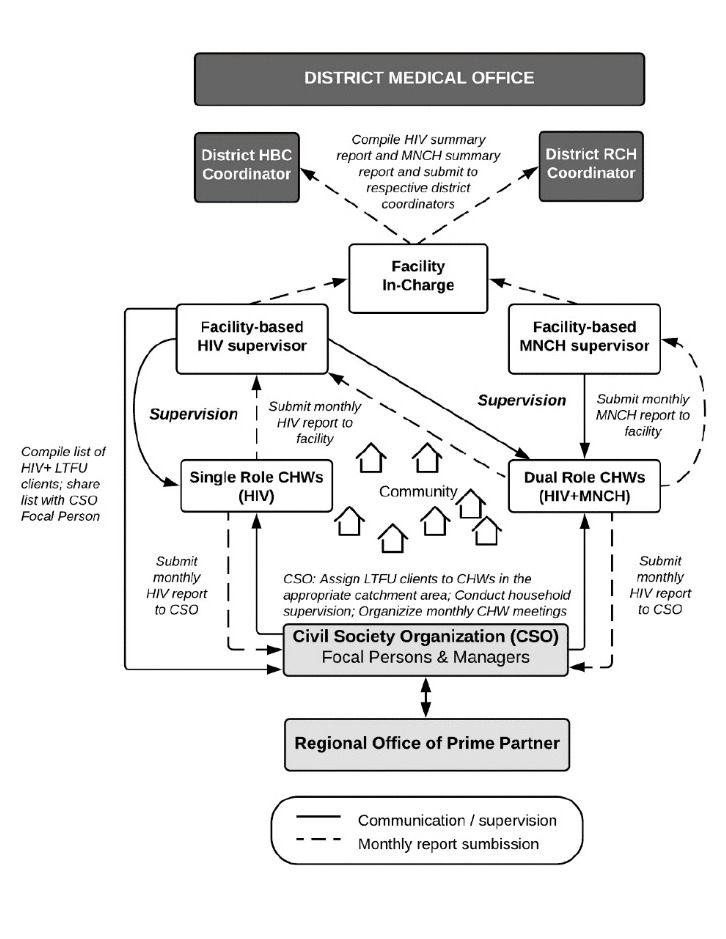
Relationship Between the Prime Partner’s Regional Office, CSO, CHWs, and Health Facility Staff for Data Reporting Flow and Supervision.
Abbreviations: HBC, home-based care; MNCH, maternal, newborn, and child health; CHW, community health worker; CSO, civil society organization; LTFU, loss to follow-up.

Supervision and management systems for the integrated model were largely separate by HIV and MNCH domains, with infrequent intersection between facility supervisors, CSOs and the prime partner. The data reporting flow and supervision processes are depicted in Figure 2. Most dual role CHWs report to both HIV and MNCH supervisors, usually located at the same facility, while some report to HIV and MNCH supervisors at separate facilities. At some facilities, the MNCH-focused supervisor provides support on both HIV and MNCH tasks, a scenario observed at about 25% of facilities sampled for dual role CHW IDIs (Supplementary file 2). A HBC focal person from the CSO also provides supervisory support to CHWs for HIV-related tasks. All CHWs submit a monthly HIV summary report to their facility-based HIV supervisor, with a copy also to their CSO-based HBC focal person. Additionally, dual role CHWs submit a monthly MNCH summary report to their facility-based MNCH supervisor, but *not* to the CSO.

### Feasibility: Role Expansion and Workload

Perspectives on CHW role expansion were assessed to understand task planning, prioritization, integration strategies, and choices made by dual role CHWs in carrying out their volunteer HIV and MNCH duties. The relationship between the additional MNCH workload and HIV role maintenance was also considered.

### Task Planning and Integration 

Dual role CHWs frequently mentioned the importance of “timetables” in planning their schedules to ensure effective management of both HIV and MNCH tasks. CHWs often scheduled HIV and MNCH home visits on different days of the week, revealing many CHWs were not conceptualizing a fully “integrated” program. However, dual role CHWs recognized there were opportunities for combining HIV and MNCH services, as illustrated by this CHW:

*“Yes, in order to be able to work efficiently the [HIV and MNCH] days should be different, unless when the same patient falls into both categories. For example, when the pregnant mother is also HIV-positive. So, here you kill two birds with one stone”* (Dual Role CHW, Male, Dispensary, Kilolo).

Several reasons for scheduling HIV and MNCH tasks on separate days were explained. That MNCH visits can require a flexible schedule responsive to the changing health needs of the mother. For example:

*“For the pregnant mothers, I am supposed to visit them when they have 3 months, eight months, and then after giving birth. But it depends, because sometimes there are emergencies, that means I can visit her any time… but for the case of HIV infected people, I visit them at least twice a month on a proper schedule. There is timetable for visiting pregnant mothers and infant children in order to know their development and give them advice”* (Dual Role CHW, Female, Health Center, Iringa Rural).

During MNCH training, dual role CHWs were instructed to plan for HIV and MNCH activities on separate days for operational reasons: Carrying both HIV and MNCH registers is burdensome and lesson plans and educational messaging are different for the two services. For example:

*“When you serve people in the community, you need to sort your patients into categories... We discouraged them to provide both HIV/AIDS and MNCH services concurrently because it would be impossible for the same HBC to carry and correctly fill out the registers for HIV/AIDS patients and for MNCH services... We discourage them to do so because there are those who can handle the two services well and those who cannot afford to mix the two services at once”* (Regional Director, Female, Prime Partner).

### Task Prioritization

Interviews explored whether dual role CHWs balanced HIV and MNCH components or prioritized one of the services over the other. Some facility supervisors felt that CHWs balanced the tasks well, and about half of dual role CHWs stated there was equal weighting of the two tasks. Respondents recognized both HIV and MNCH services were critical to the community’s health, and neither could be neglected:

*“It’s very challenging but we have to manage both of them because you can’t take care of one client and leave another. All are human beings”* (Dual Role CHW, Female, Dispensary, Kilolo).

In contrast, among the dual role CHW respondents that prioritized MNCH, they cited several reasons. MNCH clients were often perceived to have more urgent acute complications. The increased availability of antiretroviral treatment, ensuring better health outcomes for HIV patients, has progressively shaped views of HIV as a chronic health condition. Therefore, the stage of the HIV epidemic may have contributed to CHW perceptions that HIV patients required less urgent care. Additional reasons to prioritize MNCH included the impact on two lives (mother and child) and the perception that pregnant and postpartum women were more in need of education.

There was some indication that dual role CHWs considered HIV tasks as “common” or “routine,” whereas MNCH tasks were “novel”, another possible driver of prioritization:

*“I always begin with MNCH because I am already used to providing HBC services”* (Dual Role CHW, Female, Dispensary, Iringa Rural).

In addition, staff at the CSO recognized that task prioritization could be problematic:

*“It can reach a point when HBCs would find themselves focusing on one aspect of the services over the other while he or she is supposed to provide both services. This can be a challenge because prioritizing one side of the service may lead the other segment to slow down. But I think most HBCs are doing well”* (HBC Focal Person, Female, CSO, Iringa Rural).

### Workload and HIV Role Maintenance

Geographic catchment areas did not change after HIV and MNCH integration, but the number of target households increased because most homes have children under-5 years. Many respondents discussed this issue in terms of workload. The MNCH service:

*“…Requires them [dual role CHWs] to visit every house to determine who is a child and who is not and to know which household has an infant child, which household has a pregnant woman or which household has a [health] problem”* (MNCH Supervisor, Female, Dispensary, Kilolo).

Nearly all respondents discussed the added workload of MNCH but believed HIV responsibilities had not been adversely affected. Dual role CHWs explained that HIV household visits required less time due to improvements in health status and shifting many lessons to group support meetings.

*“What helps me now is that at the inception of the HBC service most of the HIV victims were bedridden but after the provision of services and education, and due to the patients’ readiness to follow the directives, their health has become stable. This makes it possible for me because instead of going to visit them in their households we now meet during group activities”* (Dual Role CHW, Male, Dispensary, Kilolo).

Dual role CHWs explained they could maintain their original HIV task responsibilities, but this came at a cost in reduced personal time:

*“I use much of my time volunteering. So, you decrease your income because sometimes you use the time that you were supposed to go to cultivate in the evening for visiting your clients to provide services. Sometimes you spend the whole day visiting patients because the village is large and there are different places to visit”* (Dual Role CHW, Male, Dispensary, Kilolo).

The prime partner also expressed concern that the extra burden of MNCH workload could reduce time for personal responsibilities and other income generating activities:

*“Giving the HBC too many responsibilities will make them not to have enough time to take care of their other activities…we do not want to take too much time from them because at the end of the day HBCs are not salaried, they only receive a stipend”* (Regional Director, Female, Prime Partner).

Similarly, some staff from the CSOs expressed concern with the added MNCH workload, suggesting that some CHWs were not able to handle existing HIV responsibilities:

*“When I look even at the normal HBC activities of caring for people with HIV/AIDS I can see that they [providers] are already overwhelmed, and when you add other responsibilities maybe only those who can really work hard will be able to handle it. But with added responsibilities most HBCs are really struggling as you have witnessed yesterday that some HBCs had not completed the tasks”* (Program Manager, Female, CSO, Kilolo).

Acceptability of Dual Role CHW Model

### CHW Satisfaction With Expanded Role

The intersection of HIV and MNCH services is critical in communities with elevated HIV prevalence and high maternal and child morbidity and mortality, “*Because even the person living with HIV/AIDS might need to make babies*” (HBC Focal Person, Female, CSO, Kilolo). Many dual role CHWs drew work satisfaction through their new ability to help improve maternal and child health in their community, in addition to their longstanding HIV role. For example:

*“Firstly, we were doing [our] work we could see the problems facing pregnant women but we could not be able to do anything though our intention was one [to help women]. We were dealing with AIDS only and the other problems we could see them but we could not tackle them but now after being trained it has been easy for us to help people”* (Dual Role CHW, Male, Dispensary, Kilolo).

The additional MNCH responsibilities were also associated with increased community recognition and a sense of feeling valued:

*“My experience has improved; first I have become known to many, and also, they have recognized me as a health leader in the ward”* (Dual Role CHW, Male, Dispensary, Kilolo).

Education and acquisition of new skills was also acknowledged as an advantage of the integrated model, both personally and for the community:

*“Most HBCs [providers] take the added responsibility as an opportunity to expand their knowledge and scope. Also, they enjoy the fact that their community acceptance has increased now that they are no longer seen as people providing exclusive HIV/AIDS care alone”* (Regional Director, Female, Prime Partner).

Some CHWs also spoke of their duty or obligation to perform the extra MNCH responsibilities because they had been selected or nominated for that purpose:

*“To some extent this is a volunteering work and remember the community has appointed you. Therefore, you are supposed to work for the community and as a volunteer. So, you work by remembering that the community has trusted you”* (Dual Role CHW, Female, Health Center, Iringa Rural).

While the contribution to society was a major driver of work satisfaction, respondents also mentioned the issue of increased workload coupled with a low stipend as de-motivating:

*“The HBCs [providers] have received it very positively. Yet, they complain that the workload is too big, and they find it hard to handle both responsibilities. Despite the work overload that they complain about, they provide the required services very well in caring for people living with HIV/AIDS as well as MNCH”* (MNCH Supervisor, Female, Dispensary, Kilolo).

Some CHWs expressed dissatisfaction with the dual role, primarily linked to the stipend issue. There was an expectation that an increase in workload and responsibilities should be accompanied by a commensurate increase in monthly stipend. The modest increase in monthly stipend from 35 000 to 40 000 Tanzanian Shillings (~US$17 to US$20) was for *all* CHWs, irrespective of single or dual role responsibilities. Some CHWs associated the monthly stipend only with their HIV role, perceiving their MNCH role as completely voluntary since it came with no additional stipend:

*“I am satisfied though we gain nothing [not] even a soap, in the first job we are given Tshs 35 000/= after a couple of months but this job we just only volunteer. We are satisfied and at the beginning we were told it is volunteering job, so we are just working with the hope that [one day] they will think of us. Unfortunately, as time goes on there is nothing new, so we just know it is volunteering job”* (Dual Role CHW, Female, Dispensary, Kilolo).

Supervisors and CSO staff reiterated a need for increased incentives to improve program acceptability:

*“Honestly, the ones I heard were complaining about a huge workload and low pay. They complained that they had a huge workload, and we then asked them what if we increased their remuneration, and they replied, yes, that would help them find alternative ways such as find help to compensate for their other income generating activities like farm work, which they forsake when concentrating on their HBC engagements”* (Program Manager, Female, CSO, Kilolo).

### One Role vs. Two Roles for Volunteer CHWs

Respondents were asked about whether CHWs should have 1 or 2 roles. Many dual role CHWs were satisfied with their combined HIV and MNCH responsibilities, while some preferred to have only one role. These nuanced views on multiple responsibilities are highlighted by the following excerpts:

“*…I would choose to remain as a community health worker [MNCH], only that. You know, in the past HIV was something strange but now we are educated or our HIV clients are educated… but the maternal, infant and children healthcare is new and that is why if I am to decide I would like to serve women and children in order to reduce deaths of women and children through education. People are steeped in HIV education in that it has become something normal” (Dual Role CHW, Male, Dispensary, Kilolo). *

*“I prefer AIDS…Because I am used to this work and I have been doing it for quite some time now and people know me as a home-based care health service provider”* (Dual Role CHW, Female, Health Center, Iringa Rural).

Single role CHWs were interested to receive the training on MNCH, with some stating their communities were at a disadvantage by not yet having a dual role CHW. However, they acknowledged the extra MNCH workload could be challenging and they expressed uncertainty about the time commitment.

Facility supervisors were mixed in their opinions about whether the HIV and MNCH roles should be integrated by CHWs. Some cited benefits to patients of providing both services together but questioned the efficiency of balancing two responsibilities. In contrast, CSO staff primarily thought HIV and MNCH roles should remain separate, while staff from the prime partner supported integration:

*“The combined services should be separated so as to increase efficiency because most HBCs are primary school leavers and they have other work to do, including farm work, to raise their income, they generally have so much in their hands… Therefore, I generally think that the services need to be separated so as to achieve better results”* (Program Manager, Female, CSO, Kilolo).

*“I think that the same volunteer can act both as CHW and an HBC and be able to follow up on the patients. The volunteers only need to be provided with tools for them to do the job because that is one of the major challenges”* (HIV Technical Officer, Female, Prime Partner).

## Discussion

**Figure 3:**
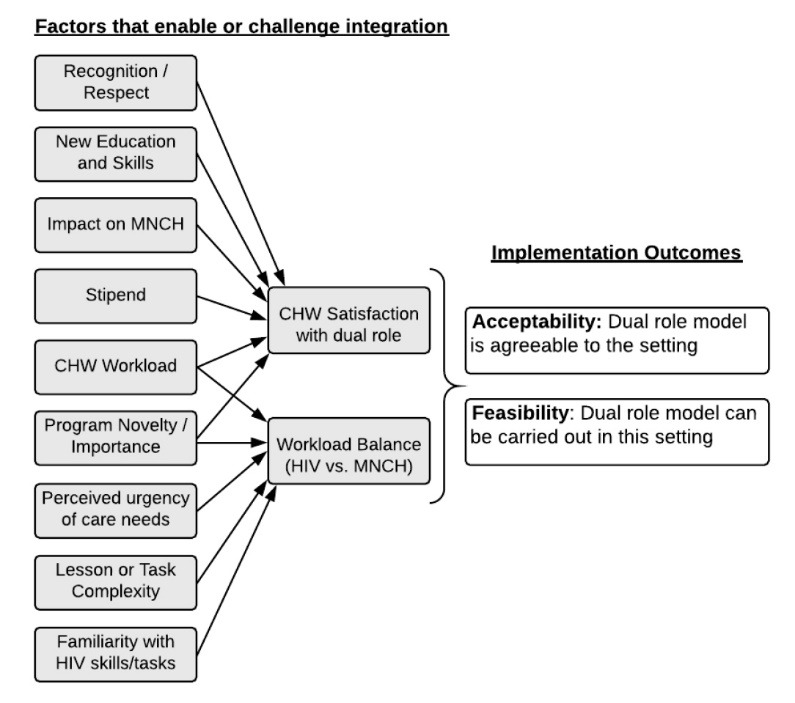
Drivers of Feasibility and Acceptability of the HIV-MNCH Dual Role Model. Abbreviations: MNCH, maternal, newborn, and child health; CHW, community health worker.

This case study describes program modifications to an established HIV-focused volunteer CHW program to include MNCH responsibilities. IDIs with a broad range of program implementers helped identify factors that supported or challenged integration efforts. CHW satisfaction and workload balance appeared linked to feasibility and acceptability of the integrated model (Figure 3). Sources of satisfaction with dual responsibilities included new education, increased community respect, perceived importance of the MNCH tasks, and improved MNCH outcomes. These results are consistent with a recent qualitative evidence synthesis of implementation barriers and facilitators of lay health worker programs to improve MNCH access, which found key motivating factors to be altruism, social recognition, and increased knowledge.^28^ Most CHWs attempted to balance HIV and MNCH responsibilities. However, some CHWs prioritized MNCH responsibilities, citing increased community recognition and perceived importance and urgency of the work. Workload balance also sometimes shifted toward MNCH responsibilities, partially due to lengthier household visits and the urgency of MNCH conditions, in contrast to the perceived stabilization of HIV needs in the community and overall long-term experience of CHWs in providing home-based HIV care. Through triangulation with routine quantitative data on HIV and MNCH visits performed by CHWs, findings presented in an associated paper also suggest the addition of MNCH tasks was generally feasible and confirm dual role CHWs were largely able to maintain their HIV workload over time.^29^

Several findings in this study suggest the program was only “partially” integrated. Most notably, dual role CHWs often carried out HIV and MNCH responsibilities on separate days, so that tasks remained in silos, rather than integrated. If clients required both HIV and MNCH services, some CHWs reported providing both services during a single household visit. That most CHWs delivered HIV and MNCH education on separate days is likely attributable to the planning guidance received during MNCH training. In addition, the HIV and MNCH domains remained separate regarding systems of supervision, data reporting, and management. Interviews revealed a complex system that included different lines of data reporting for HIV and MNCH services, separate facility-based supervisors for HIV and MNCH, and support from the CSOs that was mostly HIV-focused (Figure 2). These findings suggest that future program design should consider higher level systems integration in supporting community-level integration.

Realistic workloads can help prevent CHW burnout and sustain motivation. Studies examining extra time requirements for additional CHW tasks have reported mixed results. In Bangladesh, CHWs spent significantly more hours per week volunteering when their workload expanded to include treatment of severe acute malnutrition.^8^ However, in Rwanda, adding family planning to existing CHW responsibilities did not significantly change reported time spent on service provision or travel.^7^ Our study did not directly measure CHW time spent volunteering, but dual role CHWs described spending 2 to 3 additional days per week performing MNCH responsibilities, on top of their HIV responsibilities. This was consistent with a recent survey in Morogoro, Tanzania which found volunteer MNCH-focused CHWs averaging 2.9 work days per week.^30^

When CHWs are responsible for more activities, role overload becomes a key risk due to the increased time and effort by volunteers.^31^ This study found similar challenges to those identified in Malawi among CHWs with expanded roles: newly learned skills were often over-emphasized and task overloading could result in CHWs being unable to fill multiple roles.^9^ The joint provision of both HIV and MNCH tasks by volunteer CHWs was feasible and generally well accepted in rural Tanzania, although challenged by workload and remuneration issues. The increased MNCH workload for the dual role CHWs did not appear to interfere with HIV responsibilities but drew time away from other income generating activities. This was similar to CHW time allocation documented in Burkina Faso, Nigeria, and Uganda, where personal time and time spent on agriculture decreased when rapid malaria testing and treatment were added to CHWs tasks.^32^ In this study, the most common recommendation was to increase the monthly stipend commensurate with added time requirements for MNCH responsibilities, as dissatisfaction with stipend levels was widespread and could challenge longer term acceptability of the integrated model. These findings fit within the larger debate regarding CHW volunteerism, where some argue the reliance on unpaid labor is unsustainable and creates unintended consequences on local communities,^33,34^ while others suggest the need for targeted alignment of incentives to the expectations within a given community context to ensure CHW performance.^35^ The Tanzanian Government’s recent policy to develop a paid, “professional” cadre of multi-task CHWs suggests a shift away from volunteer-based community health provision.^12^

### Ensuring Rigor

Steps to ensure research rigor (trustworthiness) were taken throughout the study design, data collection, and analytical phases.^36^ To improve credibility and foster reflexivity, the research team debriefed after each data collection event to discuss IDI findings and recurrent and emergent themes as well as for real-time feedback to programmatic and district management stakeholders. The iterative process of team debriefing helped reveal our own research biases evident in how questions were formulated and asked and suggested additional probes and question modification helpful for going forward. In the analysis phase, comprehensive reviews of transcripts, along with peer debriefing between coders, helped improve data credibility.^37^ One of the primary coders provided an external check on data interpretation, having not been involved in study design or data collection. A strength of this study was its multiple perspectives from respondents involved in all aspects of the integrated model in two districts, including the viewpoints of program managers and technical staff from the CSO and prime partner – an often underrepresented perspective in research on lay health worker programs.^28^ To improve dependability and confirmability, an audit trail documented all study procedures thoroughly and transparently, including decisions related to sampling, recruitment, data collection, and analysis.^38^ Through careful documentation of the implementation context, transferability of study findings to other volunteer CHW programs looking to expand roles and responsibilities was improved. In Tanzania, where roughly half of all volunteer CHW programs are either HIV or MNCH-focused, this study is highly relevant to ongoing implementation decisions, supporting HIV-MNCH integration at the community-level.

### Study Limitations

Evidence is limited to perspectives from implementers and given their potential contribution to the design of the integrated CHW model, their insight on feasibility and acceptability should be assessed with reflexivity considerations in mind. Although we did not investigate the role of program staff in designing the integrated CHW model, we did gain perspectives from CHWs and their supervisors who were not involved in program design nor in considering how to assess its feasibility or acceptability. Interviews with community members, including community leaders, would have offered additional perspective on program acceptability and should be incorporated into future studies. In addition, observation of household visits would contribute understanding of *how* dual role CHWs performed their integrated duties and contribute to measures of implementation fidelity. IDIs were conducted relatively early during the implementation phase, meaning findings do not reflect long term perceptions of the integrated model or later stage implementation outcomes, such as sustainability. A further limitation is the potential for social desirability bias, particularly among CHWs who may have felt compelled to answer favorably, and program managers who may have shared opinions they thought researchers wanted to hear. Bias was minimized through training research assistants to remain neutral, build rapport, reiterate confidentiality, and emphasize that participant responses would help in developing stronger community health systems.

## Conclusion

Findings from this study offer a nuanced portrait of the experience of volunteer CHWs and program managers regarding role expansion from HIV to MNCH. This study demonstrated that a volunteer CHW can feasibly balance the two different roles of providing HIV and MNCH health promotion in community households, but not without some challenges related to the heavier workload and incommensurate increase in remuneration. High program acceptance was found in its early implementation. Further research is needed to determine the quality of dual health promotion for HIV and MNCH domains under such an integrated approach, and what can be adapted from this model, in order to scale and sustain integrated approaches that are more responsive to community needs and CHW interests.

## Acknowledgements

We thank the late Helen Semu, Assistant Director of the Health Promotion Unit in the Ministry of Health, Community Development, Gender, Elderly and Children (MoHCDGEC) for her guidance and support throughout the Community Health Worker Learning Agenda Project (CHW-LAP). The present research was embedded within CHW-LAP, a multiyear implementation research partnership between Muhimbili University of Health and Allied Sciences (MUHAS) in Tanzania and Johns Hopkins Bloomberg School of Public Health (JHSPH), USA, which was funded through the United States Agency for International Development (USAID). We extend our gratitude to the Iringa Regional Medical Office and the Iringa Rural and Kilolo District Health Management Teams for facilitation of our data collection plans and overall interest in the research. We are grateful for the collaboration with Deloitte Consulting Limited and Christian Social Services Commission, implementers of TUNAJALI II, who provided insights on their program design and approach, and linkages to their civil society partners who were integral to facilitating data collection. Endless appreciation and thanks for the tireless work of our data collection team in Iringa: Ismail Amiri, Bernadetha Hubert, Emmanuel Maawe, Christina Maluli, Debora Mlambo, Hereswida Monyiechi, Maurus Mpunga, Annobeatrice Mville, and Zaina Sheweji. Our thanks to several additional MUHAS colleagues, including Idda Mosha who helped oversee the qualitative data collection, Patrick Kazonda who managed data entry and cleaning of the CHW demographic survey, and Aisha Omary and Mary Glory Emmanuel who provided administrative support. Thanks also to JHSPH colleague Kadia Petricca for input on the study design. Special thanks to Priscia Wanjiro of MoHCDGEC for serving as our liaison during data collection in Iringa.

## Ethical issues

The study was jointly approved for ethical clearance by the Institutional Review Boards of Johns Hopkins School of Public Health in Baltimore, MD, USA (IRB No. 00005497) and Muhimbili University of Health and Allied Sciences in Dar es Salaam, Tanzania (Ref. No. 2015-12-18/AEC/Vol. X/94). All potential study participants underwent a verbal informed consent process in Swahili using an IRB-approved consent form.

## Competing interests

Asha George serves as the Vice Chair of Health Systems Global. Asha George is supported by the South African Research Chair’s Initiative of the Department of Science and Technology and National Research Foundation (NRF) of South Africa (Grant No 82769). Any opinion, finding and conclusion or recommendation expressed in this material is that of the author and the NRF does not accept any liability in this regard.

Hawa Kadria Kisusi served as a *Senior Technical Officer – Home Based Care* at Christian Social Services Commission, the Technical Partner for TUNAJALI II.

All other authors declare no competing interests.

## Authors’ contributions

AHB and JK are the Principal Investigators of the overarching evaluation within which we designed and collected data for this study. As part of her PhD dissertation at JHSPH, KDS designed the protocol and developed interview guides with inputs from all authors. HKK provided background data and information on program context and design, which facilitated the implementation research approach. KDS and GF coordinated and oversaw qualitative data collection and management, with support from JJ who also conducted quality assurance review of interview transcripts and translations. KDS and GM coded all transcripts. KDS conducted qualitative analysis, with guidance from ASG, GF, DHP, AHB, and RM on interpretation and presentation of results. KDS wrote the first draft of the paper, with revision and inputs from ASG, DHP, AHB, GF, and RM. All authors read and approved the final manuscript.

## Authors’ affiliations

^1^Department of International Health, Johns Hopkins Bloomberg School of Public Health, Baltimore, MD, USA. ^2^Department of Development Studies, School of Public Health and Social Sciences, Muhimbili University of Health and Allied Sciences, Dar es Salaam, Tanzania. ^3^Department of Epidemiology and Biostatistics, School of Public Health and Social Sciences, Muhimbili University of Health and Allied Sciences, Dar es Salaam, Tanzania. ^4^Christian Social Services Commission, Dar es Salaam, Tanzania. ^5^School of Public Health, University of the Western Cape, Cape Town, South Africa.

## Funding

USAID through the Health Research Challenge for Impact (HRCI) Cooperative Agreement (#GHS-A-00-09-00004-00) with the Johns Hopkins Bloomberg School of Public Health (JHSPH) , Baltimore, MD, USA. The contents are the responsibility of the authors and do not necessarily reflect the views of USAID or the United States Government.

## Supplementary files


Supplementary file 1. Sequencing of Qualitative and Quantitative Data Collection Across the Mixed Methods Evaluation.

Supplementary file 2. Summary of Facility, CHW and Supervisor Characteristics Among Interview Respondents at Each Sampled Facility, by District.

## Key messages

**Implications for policy makers**

- As countries move forward with primary healthcare plans to revitalize and expand community health worker (CHW) cadres, whether CHWs should focus on one health area (“vertical” approach) or multiple health areas (“horizontal” approach) is a common policy concern where there is little data to inform choices.
- The intersection of HIV and maternal, newborn and child health (MNCH) services is critical in communities with elevated HIV prevalence and high maternal and child morbidity and mortality, but there is limited evidence around service integration at the community level. Building “people-centered” primary healthcare systems will require consideration of service integration opportunities by CHWs, where the potential benefits to both providers and clients may include increased satisfaction, improved care outcomes, reduced fragmentation of services, and cost and human resource efficiencies.
- Carefully consider opportunities for integration and evaluate realistic CHW workloads and compensation packages (monetary and nonmonetary) during policy and planning stages—a particularly relevant concern in countries expanding national multi-tasked CHW cadres, such as Tanzania.

**Implications for public**

Community health workers (CHWs) extend healthcare delivery to communities and serve a critical linkage function with health facilities. This study describes a program innovation to combine HIV and maternal, newborn and child health (MNCH) promotion and explores how CHWs manage dual role responsibilities and why it works (or not). Findings suggest MNCH promotion can feasibly be added to the workload of volunteer HIV-focused CHWs in rural Tanzania, with high program acceptance documented early in implementation. CHWs attempted to balance HIV and MNCH responsibilities; however, the extra workload took time away from other income generating activities. Understanding reasons for CHW satisfaction with the dual role model (increased respect in the community, new education and skills, and personal fulfilment from helping improve maternal and child health in their communities) and dissatisfaction (workload not commensurate with stipend) are important outputs from this study which can help policy makers and planners design more effective integrated service delivery models at the community-level.
