# Supplementary material for: "Because Even the Person Living With HIV/AIDS Might Need to Make Babies" – Perspectives on the Drivers of Feasibility and Acceptability of an Integrated Community Health Worker Model in Iringa, Tanzania
Source: Int J Health Policy Manag. 2019 Jun 11;8(9):538–49. doi: 10.15171/ijhpm.2019.38 (PMC6815988; doi:10.15171/ijhpm.2019.38)

Supplementary File 1. Sequencing of Qualitative and Quantitative Data Collection Across the Mixed Methods Evaluation

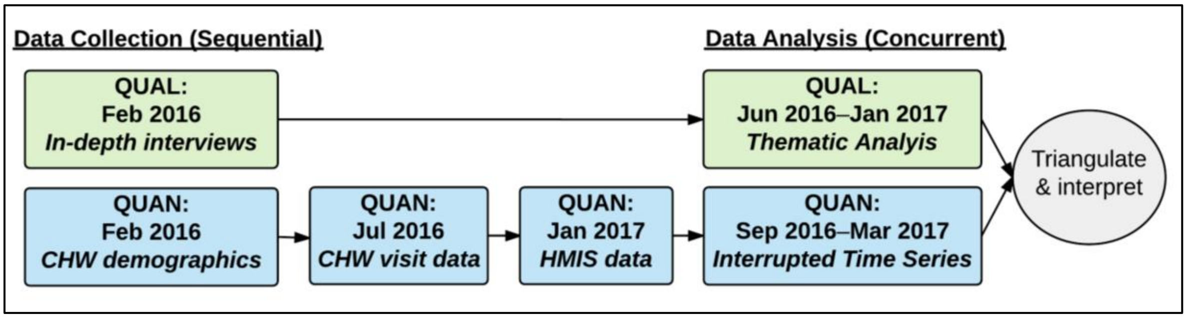

Supplement: Supplementary file 1 — Sequencing of Qualitative and Quantitative Data Collection Across the Mixed Methods Evaluation. [file ijhpm-8-538-s001.pdf]
