# Supplementary material for: "Because Even the Person Living With HIV/AIDS Might Need to Make Babies" – Perspectives on the Drivers of Feasibility and Acceptability of an Integrated Community Health Worker Model in Iringa, Tanzania
Source: Int J Health Policy Manag. 2019 Jun 11;8(9):538–49. doi: 10.15171/ijhpm.2019.38 (PMC6815988; doi:10.15171/ijhpm.2019.38)
Supplement: Supplementary file 2 — Summary of Facility, CHW and Supervisor Characteristics Among Interview Respondents at Each Sampled Facility, by District. [file ijhpm-8-538-s002.pdf]

Supplementary file 2. Summary of Facility, CHW and Supervisor Characteristics Among Interview Respondents at Each Sampled Facility, by District

Facility		HIV	Referral	Utilization / Mo. ϕ		CHW IDIs	CHW Sex		Facility-based Supervisor IDIs: Qualifications	
**ID**	**Owner**	**Services†**	**Distance‡**	**ANC1**	**Delivery**	**Type (Total IDIs)**	**#M**	**#F**	**HIV Supervisor**	**MNCH Supervisor***
**Iringa Rural Summary**			**Avg=52**	**Avg=9.2**	**Avg=9.7**	**9 Single; 8 Dual**	**6M**	**11F**	**5 HIV supervisors**	**5 MNCH supervisors**
D10	Public	RCH	40 KM	4.2	2.8	Single (2)	1M	1F	Medical Attendant	*Not Applicable*
D11	FBO	RCH	65 KM	5.1	5.3	Single (2)	1M	1F	Medical Attendant	*Not Applicable*
D12	Public	CTC/RCH	49 KM	6.0	8.9	Single (3)	2M	1F	*Unavailable*	*Not Applicable*
D13	FBO	RCH	67 KM	16.1	4.1	Dual (1)	—	1F	*None*	Nurse Midwife
D14	Public	RCH	15 KM	6.9	7.7	Dual (2)	1M	1F	*None*	Enrolled Nurse*
D15	Public	RCH	92 KM	12.2	12.9	Dual (2)	—	2F	Medical Attendant	Enrolled Nurse
HC2	Public	CTC/RCH	41 KM	12.7	25.5	Single (1)	—	1F	*Unavailable*	*Not Applicable*
HC3	Public	CTC/RCH	34 KM	11.3	10.6	Dual (1) Single (1)	1M	1F	Registered Nurse	Registered Nurse
HC4	Public	CTC/RCH	47 KM	9.8	8.8	Dual (1)	—	1F	*Not interviewed*	*Not interviewed*
HC5	Public	CTC/RCH	72 KM	8.0	10.7	Dual (1)	—	1F	Midwife	Registered Nurse
**Kilolo Summary**			**Avg=32**	**Avg=9.0**	**Avg=9.3**	**6 Single; 13 Dual**	**11M**	**8F**	**5 HIV supervisors**	**6 MNCH supervisors**
D1	Public	RCH	10 KM	4.1	4.0	Single (3)	2M	1F	Clinical Attendant	*Not Applicable*
D2	FBO	RCH	1 KM	26.0	12.5	Single (1)	—	1F	*Unavailable*	*Not Applicable*
D3	Public	RCH	41 KM	7.1	5.8	Single (2)	1M	1F	Enrolled Nurse	*Not Applicable*
D4	Public	RCH	65 KM	5.4	5.3	Dual (2)	1M	1F	*None*	Medical Attendant
D5	Public	RCH	10 KM	8.2	8.4	Dual (1)	—	1F	Enrolled Nurse	Clinical Officer
D6	Public	RCH	26 KM	2.8	1.2	Dual (1)	1M	—	Medical Attendant	*None*
D7	Public	CTC/RCH	75 KM	6.2	6.7	Dual (3)	3M	—	*Unavailable*	Midwife
D8	FBO	CTC/RCH	21 KM	7.5	12.3	Dual (2)	1M	1F	*None*	Enrolled Nurse*
D9	Public	CTC/RCH	19 KM	17.9	24.1	Dual (2)	1M	1F	*None*	Registered Nurse*
HC1	Public	CTC/RCH	55 KM	4.8	12.6	Dual (2)	1M	1F	Medical Attendant	Enrolled Nurse
**Combined Summary**			**Avg=42**	**Avg=9.1**	**Avg=9.5**	**15 Single; 21 Dual**	**17M**	**19F**	**10 HIV supervisors**	**11 MNCH supervisors**

Abbreviations: D=Dispensary; HC=Health Center; KM=kilometers; M=Male; F=Female; FBO=Faith Based Organization
† HIV Care Services: CTC=Care & Treatment (adult & pediatric); RCH=Reproductive & Child Health (PMTCT, often including Option B+)
‡ Referral distance is the number of kilometers to the nearest hospital (either the district hospital or regional referral hospital) and is an indication of the remoteness of the facility
ϕ Service utilization is the average monthly number of 1st ANC visits and average monthly number of facility deliveries from Tanzania's 2015 DHIS2
Not Applicable indicates there was no MNCH supervisor because the facility does not have any dual role CHWs
Unavailable indicates the interview could not be scheduled (i.e. the supervisor was travelling off-site or on-leave)
None indicates the facility had no staff trained to provide CHWs with either HIV or MNCH-specific supervision
*In some cases, the MNCH supervisor provided CHWs with supervision for <u>both</u> HIV and MNCH (the case for 3 of the 11 MNCH supervisors interviewed)
Not interviewed indicates the HC4 facility was not visited for HIV and MNCH supervisor interviews; instead, the IDI with the dual role CHW occurred at the nearby D10 facility
